# An Uncommon Correlation of Rheumatoid Arthritis and Lupus Nephritis: A Case Report on the Unusual Progression of Lupus Nephritis

**DOI:** 10.7759/cureus.27620

**Published:** 2022-08-02

**Authors:** Lyna C Lam, Vinita D Yadav, Victor J Mihal

**Affiliations:** 1 Medicine, Edward Via College of Osteopathic Medicine, Boston, USA; 2 Family Medicine, Sentara Halifax Family Medicine, Boston, USA

**Keywords:** systemic lupus erythematosus, mixed connective tissue disease, nephrotic, lupus nephritis, rheumatoid arthritis

## Abstract

Systemic lupus erythematosus (SLE) is an autoimmune disease characterized by chronic, widespread inflammation and multisystem organ damage. A serious complication of SLE is damage to the kidneys, which is called lupus nephritis (LN). LN typically manifests after five years post the diagnosis of SLE. However, in this case report, the authors present a 46-year-old female with no previous history of SLE, but rather a history of rheumatoid arthritis (RA) and mixed connective tissue disease (MCTD) with the rapid progression of shortness of breath and lower extremity edema in four months’ time. Upon further workup, large amounts of protein were found in the urine, consistent with nephrotic syndrome, and renal biopsy confirmed lupus nephritis. The patient ultimately achieved symptomatic relief with Benlysta® (belimumab), a recombinant human immunoglobulin G1λ (IgG1λ) monoclonal antibody. This report aims to highlight this unique presentation of both SLE and RA and bring awareness to and facilitate the early diagnosis and treatment of LN, thus mitigating permanent kidney damage.

## Introduction

Systemic lupus erythematosus (SLE) is an autoimmune disease characterized by chronic, widespread inflammation, and multisystem organ damage. African-Americans are more frequently affected than Caucasians [1]. Women of childbearing age account for 90% of cases, although men have a more severe disease [2]. The etiology of this condition remains unknown, however, the causative factors include the loss of self-tolerance in individuals with a genetic predisposition following exposure to environmental triggers [3]. The pathophysiology of SLE involves autoantibody production, deposition of immune complexes, complement activation, and accompanying tissue destruction/vasculitis. Diagnosis is based on the 2019 European League Against Rheumatism (EULAR) and American College of Rheumatology (ACR) classification criteria, which consists of a list of clinical and immunologic criteria, which each equates to a certain number of points [4]. A patient must have at least one clinical criterion and a total score of 10 points, later described in detail.

Specifically, lupus nephritis (LN) results from the deposition of immune complexes in the glomeruli of the kidneys (distinctively subendothelial, subepithelial, and/or mesangial). The immune complexes further trigger an inflammatory reaction with cytokines, oxidative injuries, and the consumption of complement. Ongoing chemokine-mediated inflammation further induces the activation and proliferation of fibroblasts alongside an increased synthesis of extracellular matrix [1]. This process causes severe damage to the kidneys, eventually leading to nephrotic disease. This is characterized by symptoms consisting of weight gain, anasarca, decreased urination, the foamy appearance of urine, and high blood pressure [5].

Rheumatoid arthritis (RA) is also a chronic inflammatory autoimmune disease, notably of the synovium of joints, causing damage to cartilage and bone. Age of onset is typically between 20 and 40 years and is more common in women than men. RA is diagnosed clinically, including inflammatory arthritis of multiple joints and symptoms lasting at least six weeks, and by serology, including elevated erythrocyte sedimentation rate and c-reactive protein and a positive serum rheumatoid factor or anti-citrullinated peptide antibodies [6].

Lastly, mixed connective tissue disease (MCTD) is a rare autoimmune disease diagnosed by the overlapping features of at least two connective tissue diseases, including SLE, scleroderma, RA, polymyositis, and/or dermatomyositis. Although there is no clear etiology for MCTD, the environmental exposure of genetically predisposed individuals is a likely risk factor. MCTD may initially present with nonspecific findings such as low-grade fever, arthralgia, and myalgia. As the disease progresses, it may begin to affect any organ system. The presence of anti-U1-ribonucleoprotein (anti-U1-RNP) is a key laboratory finding in MCTD and is believed to play a major role in its pathophysiology. Diagnosis can be made by using the Alarcon-Segovia criteria, which include a high anti-U1-RNP titer ≥ 1:1600 and three or more of the following clinical exam findings: edema of the hands, Raynaud’s, synovitis, myositis, and sclerodactyly [7].

It is said that with one autoimmune connective tissue disease, it is highly likely to have others, as described by the diagnosis of MCTD. However, the correlation between SLE and RA is unknown. One study performed in 1987 tested the relationship between the two diseases. The Sydney University Rheumatology Department along with The Royal North Shore Hospital of Sydney reported 11 patients with classical RA who subsequently developed SLE. They discovered that there are many similarities between the two diseases, including overlapping antibodies, thus making it difficult to distinguish the diagnosis as SLE or RA [8]. Another study from 2009 studied the prevalence of SLE features (as defined by the 1982 American College of Rheumatology criteria) in an incidence cohort of patients with RA. After following this cohort of 603 incident RA subjects for 25 years, 54.5% of the cohort developed three SLE features. However, interestingly, only 1.5% of the RA subjects had a physician diagnosis of SLE [9]. Thus, according to these studies, the two diseases may, more often than not, coexist together by chance but evade physician diagnosis.

## Case presentation

Consent was acquired from this patient to retrospectively review her case and clinical management protocols. A 46-year-old African American woman with a past medical history of rheumatoid arthritis (diagnosed 6-7 years ago) managed on Xeljanz® (tofacitinib), mixed connective tissue disease, neuropathy, gastritis, anemia secondary to iron deficiency and chronic disease, and hypertension presented to her primary care provider (PCP) with the chief complaints of shortness of breath and peripheral edema. Her past surgical history includes bilateral tubal ligation. The patient’s family history was notable for diabetes, hypertension, stroke, and bipolar disorder. She had no history of smoking or alcohol use.

The patient described new-onset shortness of breath with chest pain and worsening of her long-standing bilateral lower extremity edema. On physical exam, the patient was in no acute distress. Her weight was 87.1 kg, an increase from her baseline weight of 70.8 kg (noted 4 months prior to admission). Her blood pressure was elevated at 146/92. Other vital signs were within normal limits. The heart rate was normal, and the heart rhythm was regular. Breath sounds were normal. The abdomen was not distended. Edema was present in the lower legs bilaterally. She was evaluated for pulmonary embolism, pneumonia, and chronic heart failure with a computed tomography angiogram, complete blood count (CBC), complete metabolic panel (CMP), thyroid stimulating hormone (TSH), magnesium, D-dimers, troponin, brain natriuretic peptide (BNP), and chest X-ray. The patient was referred to cardiology for further evaluation of possible chronic heart failure with cardiac stress tests and an echocardiogram. She was started on Lasix® (furosemide), and Coreg® (carvedilol) was increased to 6.25 mg twice daily to help improve her symptoms. One month later, Coreg® was increased to 12.5 mg twice daily for better blood pressure control, and Zaroxolyn® (metolazone) 2.5 mg was initiated to further optimize diuresis. Two and a half months later, she presented to her PCP for routine follow-up. She presented with oliguria and worsening peripheral edema. CBC, CMP, and BNP were ordered and revealed worsening kidney function. She was referred to nephrology.

Five days later and prior to the nephrology appointment, she presented to the emergency department (ED) with worsening edema which had progressed to her abdomen and worsening shortness of breath. In the ED, CBC, CMP, troponin, BNP, electrocardiogram (ECG), and chest x-ray were ordered. Of note, the CMP revealed an elevated creatinine (2.0 mg/dL), elevated blood urea nitrogen (46 mg/dL), low albumin levels (1.8 g/dL), and her urinalysis was positive for protein (> 500 mg/dL). These results were suggestive of acute kidney injury secondary to nephrotic syndrome. She was admitted to inpatient service for further workup and testing, and nephrology was consulted. Serologic testing revealed positive extractable nuclear antigen ribonucleoprotein (ENA RNP), anti-Smith antibody, chromatin antibody, and anti-double stranded deoxyribonucleic acid antibody along with low total complement (CH50), and complement components 3 and 4 (C3 and C4). Given her history of rheumatoid arthritis and mixed connective tissue disease, the clinical presentation of nephrotic syndrome, and positive serology, lupus nephritis was high on the differential.

She was referred to radiology to obtain a kidney biopsy. The sections obtained consisted of predominantly medulla and a small portion of renal cortex with an attached fibrous capsule containing a single globally sclerosed glomerulus. 

Sections of the specimen submitted for conventional light microscopy were stained with hematoxylin and eosin, periodic acid-Schiff, periodic acid-methenamine silver, and Masson’s trichrome stains (all with appropriate positive controls). There was focal interstitial fibrosis and tubular atrophy with associated interstitial inflammation. Many tubular segments contained protein absorption droplets. Rare arterioles, possibly due to angiogenesis, were without significant pathologic alterations. No arteries were available for evaluation. Evaluation of the renal cortex biopsy was limited due to the lack of glomeruli in the sample submitted.

**Figure 1:**
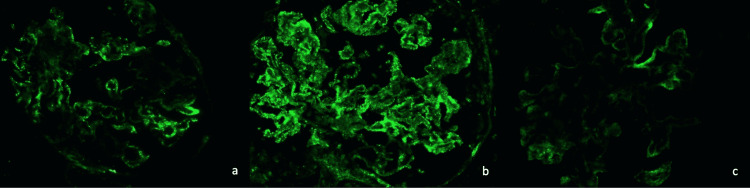
Immunofluorescence staining of renal biopsy in order (from a to c): Immunoglobulin M, Immunoglobulin G, Immunoglobulin A

**Figure 2:**
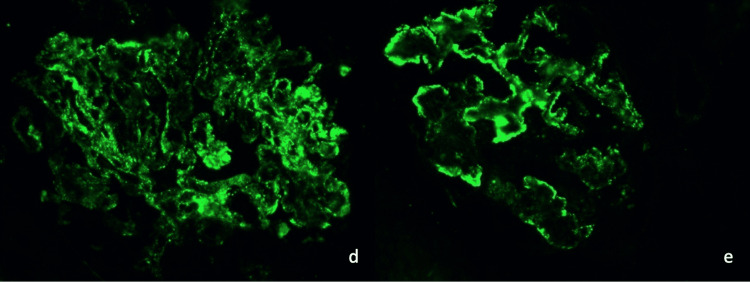
Immunofluorescence staining of renal biopsy in order (from d to e): complement component 3 (C3) and complement component 1q (C1q) “Full house” immunostaining can be seen in the patient’s renal biopsy images due to positive staining in three immunoglobulins, C3 and C1q, and is very characteristic of lupus nephritis.

Immunofluorescence microscopy was performed on frozen sections stained with fluoresceinated antisera to human immunoglobulin G antibody (IgG), immunoglobulin A (IgA), immunoglobulin M (IgM), complement component 1q (C1q), C3, albumin, fibrinogen, and kappa and lambda immunoglobulin light chains and was graded on a scale from 0 to 4+. Sections stained with IgM, IgG, IgA, C1q, and C3 immunofluorescence can be found in Figure 1 and Figure 2.

Examination of tissue sections showed a renal cortex containing 14 glomeruli, of which three were globally sclerosed. Glomeruli appeared hypercellular. A more detailed evaluation was precluded due to a significant freezing artifact. The glomeruli showed granular mesangial and capillary wall staining for IgG(ab) (4+), IgA (trace to 1+), IgM (1+), C3 (4+), C1q (4+), kappa light chains (4+), and lambda light chains (4+). There was no significant glomerular staining for fibrinogen. Arteries and arterioles showed granular staining of IgG(ab) and C3 (each 1+). Tubular reabsorption droplets stained for albumin. There was no significant staining of the tubular basement membranes or interstitium for any of the tested immune reactants. Overall, the immunofluorescence findings of “full house” staining were consistent with lupus nephritis, most likely proliferative with additional features of membranous.

Several weeks later, the patient was readmitted, and rheumatology was consulted. The following treatments were initiated: IV Eurolupus® (cyclophosphamide) infusion every two weeks for a total of six doses, Plaquenil® (hydroxychloroquine) 200 mg once a day, and a prednisone taper starting at 60 mg. Due to a lack of symptomatic improvement with Eurolupus® (cyclophosphamide), it was discontinued after three weeks and replaced with Cellcept® (mycophenolate mofetil) 500 mg tapered up to three tablets twice daily and Benlysta® (belimumab) 400 mg every seven days for four doses and then 200 mg every seven days. This dual therapy treatment plan was based on the Belimumab International Study in Lupus Nephritis (BLISS-LN), which found significant improvement in kidney responses (primary efficacy renal response and complete renal response) as compared to standard treatment (Eurolupus® or Cellcept®) alone [10]. At follow-up two months later, her condition had improved dramatically with essentially complete symptom resolution and a return to her baseline weight.

## Discussion

The patient described here reported the cardinal symptoms of nephrotic syndrome, which consists of a high level of proteinuria (> 3.5 g/24 hrs), hypoalbuminemia (< 3.5 g/dL), and peripheral edema [5]. The differential diagnosis of the nephrotic syndrome includes many etiologies such as minimal change disease, focal segmental glomerulosclerosis, membranous nephropathy, amyloidosis, diabetic glomerulonephropathy, and diffuse proliferative glomerulonephritis. Her symptoms of shortness of breath and chest pain in addition to bilateral lower extremity edema also led to consideration of congestive heart failure (CHF), acute myocardial infarction, deep venous thrombosis (DVT), hypothyroidism, and pneumonia. For CHF, the physical exam was negative for jugular venous distention, ECG was within normal limits, and the echocardiogram and nuclear stress test revealed normal ejection fractions and no acute findings. For acute myocardial infarction, ECG revealed no ST elevation or any ST changes. For DVT +/- pulmonary emboli, the D-dimer was positive, but the lower extremity venous duplex scan revealed no thrombi, and pulmonary emboli work-up was not pursued due to a low Well’s score. For hypothyroidism, TSH was within normal limits. For pneumonia, white blood cell count was within normal limits, and chest X-ray revealed cardiomegaly, but no consolidation.

**Figure 3:**
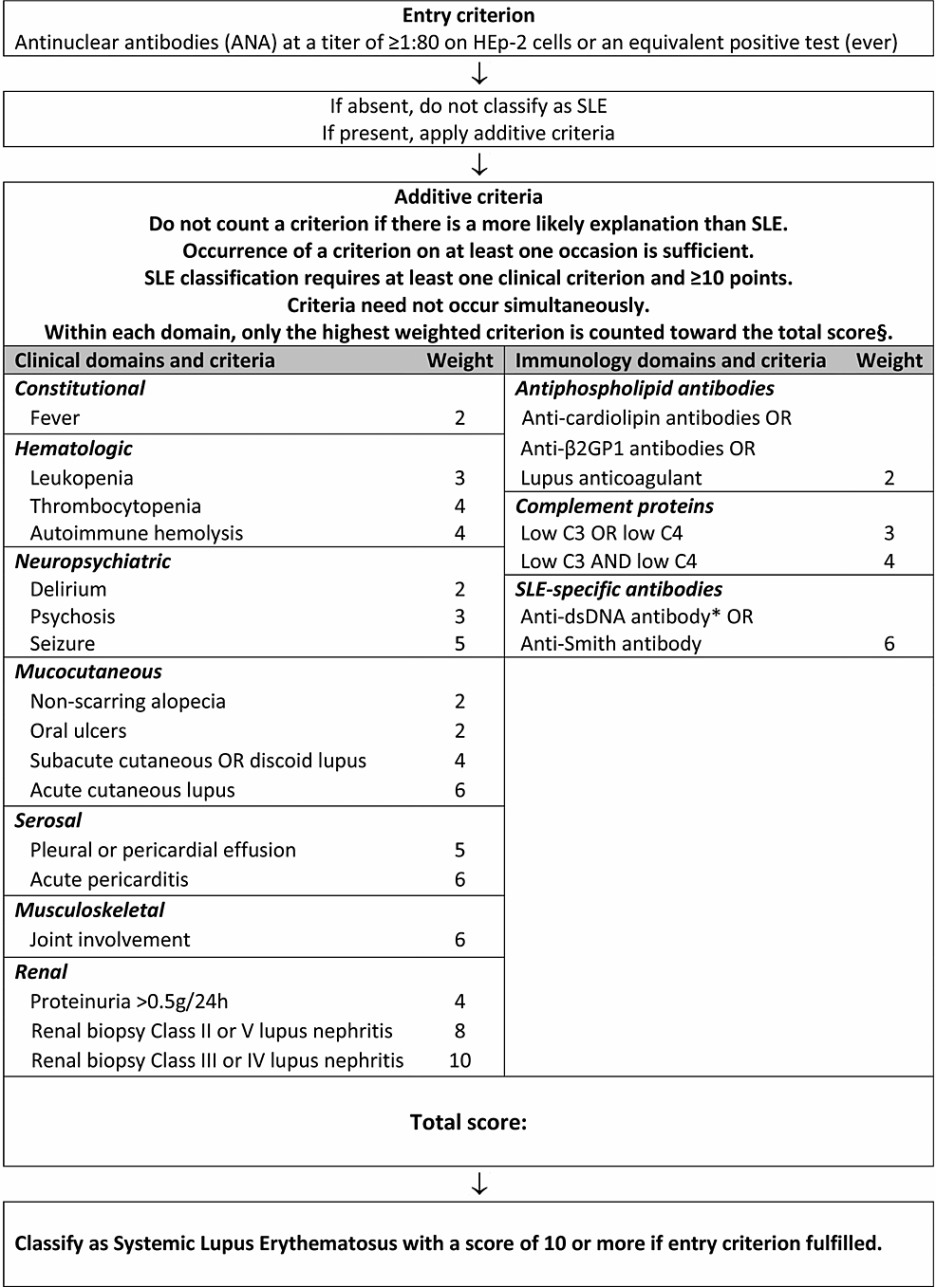
Diagnosis of systemic lupus erythematosus (SLE) can be done by using the 2019 European League Against Rheumatism (EULAR) and American College of Rheumatology (ACR) classification criteria, which contains a list of clinical and immunologic factors and corresponding points A total score of 10 or more is needed to diagnose SLE. Aringer M, Costenbader K, Daikh D, et al.: 2019 European League Against Rheumatism/American College of Rheumatology Classification Criteria for Systemic Lupus Erythematosus. Arthritis & Rheumatology. 2019, 71:1400-1412. 10.1002/art.40930

In Figure 3, the 2019 EULAR and ACR classification criteria are detailed and may serve as a tool in the diagnosis of SLE and thus LN [4].

Since the 1987 case report documenting 11 cases of RA patients who subsequently developed SLE, the overlapping antibodies found between the two diseases led researchers to believe that SLE and RA more often coexisted together by chance instead of due to a common etiology [8]. However, there is ongoing research regarding potential connections between SLE and RA. Similar phenotypic characteristics, such as joint involvement, systemic features, leading causes of mortality, autoantibody production, and response to treatments, between SLE and RA have suggested the possibility of common etiologies. The leading cause of death in patients with and without RA is cardiovascular disease (CVD). Patients with RA have a significantly higher-almost two-fold risk of developing CVD as compared to the general population. This increased risk is attributed to the systemic inflammation caused by RA and the broad use of glucocorticoids by RA patients. The systemic inflammation facilitates the development of accelerated atherosclerosis, and glucocorticoid use is associated with insulin resistance, hypertension, obesity, hyperlipidemia, and diabetes mellitus, which are all risk factors for CVD [11]. Similarly, CVD is one of the leading causes of mortality in patients with LN since vascular complications are exacerbated by renal failure secondary to LN [12].

In terms of pathogenetic similarities, antinuclear antibodies, which are a distinguishing serological marker for SLE, are more frequently positive in RA patients as compared to the general population [13]. At a cellular level, nitric oxide (NO) production by monocytes is significantly increased in SLE directly leading to lymphocyte mitochondrial dysfunction. Similarly, NO production is also notably increased in the inflamed synovium of RA patients [14]. Genotypically, research has found that human leukocyte antigen DRB1 (HLA-DRB1), protein tyrosine phosphatase non-receptor type 22 (PTPN22), signal transducer and activator of transcription 4 (STAT4), tumor necrosis factor alpha-induced protein 3 (TNFAIP3), fragment crystallizable gamma receptor 2A (FCGR2A), positive regulatory domain zinc finger protein 1 (PRDM1), interferon regulatory factor 5 (IRF5), Phox homology domain containing serine/threonine kinase-like (PXK), B lymphocyte kinase (BLK), and ubiquitin-conjugating enzyme E2 L3 (UBE2L3) are loci that show overlap between SLE and RA. All of these loci are involved in important autoimmunity and inflammatory pathways [13]. In summary, SLE and RA have overlapping features in regards to phenotypic characteristics, autoimmune pathogenesis, and genetic loci. Thus, it is highly possible that the overlapping phenotypic characteristics of SLE and RA may be caused by these overlapping genotypic characteristics that are implicated in the same immunomodulatory pathways.

Given the overlapping features of SLE and RA, there is a possibility of therapeutic options that can treat both conditions. BLK, one of the shared loci between SLE and RA, encodes a tyrosine kinase that is linked to the regulation of B-cell activation. B cells play a crucial role in the pathogenesis of SLE and RA. Consequently, B-cell depletion has been a successful approach in managing both diseases [13]. Benlysta® (belimumab) is a recombinant human immunoglobulin G1λ (IgG1λ) monoclonal antibody that inhibits B-lymphocyte stimulator (BLyS), and it serves as an add-on treatment to Cellcept® (mycophenolate mofetil) for the patient described in this case report. In lupus nephritis specifically, Benlysta® (belimumab), as an add-on to standard therapy (cyclophosphamide/azathioprine or mycophenolate mofetil), has been recently demonstrated to improve the primary efficacy of kidney response and overall decrease the risk of LN flare and kidney-related events or death in LN patients [10].

## Conclusions

This case report highlights an initial presentation of LN in a patient with no past history of SLE as well as a rare case of concurrent SLE and RA. Workup must be extensive to rule out several other common differential diagnoses based on clinical and immunological factors. As the first point of contact, family physicians and emergency physicians are crucial in the early identification of LN. Early identification is imperative, as a delayed diagnosis is common and exacerbates the mental and physical burden of LN. Furthermore, screening for RA and MCTD could be considered for patients who present with LN or vice versa in light of the diseases’ genotypic and phenotypic similarities. However, further research regarding the connection between these diseases would be beneficial to accelerate the early identification of their coexistence in patients as well as the development of multipurpose therapies with efficacy in treating multiple rheumatologic diseases. Ongoing research regarding Benlysta® (belimumab) as an add-on to standard LN therapy is promising and suggests a potentially significant increase in quality of life for LN patients. In light of the shared characteristics of SLE and RA, further research could reveal the potential utility of Benlysta® in also treating RA.
